# Serum Levels of IL-17 and IL-23 in Patients With Rheumatic Mitral Stenosis

**DOI:** 10.1097/MD.0000000000003562

**Published:** 2016-05-06

**Authors:** Mehmet Zihni Bilik, İbrahim Kaplan, Nihat Polat, Mehmet Ata Akil, Abdurrahman Akyüz, Halit Acet, Murat Yüksel, Ümit İnci, Fethullah Kayan, Nizamettin Toprak

**Affiliations:** From the Faculty of Medicine, (MZB, NP, MAA, HA, MY, NT), Department of Cardiology; Faculty of Medicine (IK), Department of Biochemistry, Dicle University; Gazi Yaşargil Educational and Research Hospital (AA), Clinic of Cardiology; Bismil State Hospital (UI), Clinic of Cardiology, Diyarbakir (UI); and Mardin State Hospital (FK), Clinic of Cardiology, Mardin, Turkey.

## Abstract

Rheumatic mitral valve stenosis (RMS) is a complication of rheumatic heart disease (RHD) and leads to significant morbidity and mortality. RHD is a chronic inflammatory and autoimmune disease that is associated with cytokine activities. The etiology of RMS is not fully understood yet. Interleukin (IL)-17 and IL-23 have a key role in development of the autoimmunity. The expression of these cytokines in RMS remains unclear. In this study, we investigated the serum levels of IL-17 and IL-23 in RMS patients compared to healthy subjects.

A total of 35 patients admitted to cardiology outpatient clinic between December 2014 and May 2015 who were diagnosed with RMS formed the study group. Age- and gender-matched 35 healthy subjects were included as the control group. Statistical analyses were performed using SPSS 18.0 and *P* value <0.05 was considered as statistically significant.

The patients with RMS had higher WBC count, hsCRP, systolic pulmonary artery pressure (PAPs), left atrial diameter (LAD), IL-17, and IL-23 levels compared to the control subjects. The levels of IL-17 (*P* = 0.012) and IL-23 (*P* = 0.004) were significantly higher in the RMS group. Correlation analysis revealed that IL-17 and IL-23 levels had a significant correlation with each other and with hsCRP and LAD.

We demonstrated that serum levels of IL-17 and IL-23 are significantly higher in patients with RMS compared to those of healthy subjects. IL-17 and IL-23 expression may have a possible role in inflammatory processes that result in RMS development.

## INTRODUCTION

Rheumatic heart disease (RHD) is a major cause of cardiovascular death in children and young adults in developing countries.^1^ RHD is the most serious complication of rheumatic fever which can lead to chronic valvular lesions. Rheumatic mitral valve stenosis (RMS) is the main presentation of RHD that leads to significant morbidity and mortality.^2^ Mitral stenosis usually develops as a result of persistent or recurrent valvulitis with bicommissural fusion.^3^ The etiology of RHD is not fully understood yet. Studies have shown that RHD is mediated by humoral and cellular autoimmune responses that occur as a consequence of long-term sequelae of Group A Streptococcus (GAS) infection.^4,5^ Previous studies suggest that RHD is an autoimmune disease ^6,7^ that is associated with cytokine activities.^8^ Inflammatory cytokines are key regulators in immune processes.^9^

It has been shown that interleukin (IL)-17 and IL-23 are potent proinflammatory molecules ^10^ that have important effects in mediating chronic inflammation, and in development of autoimmune diseases including multiple sclerosis, rheumatoid arthritis, systemic lupus erythematosus, asthma, psoriasis, and many other autoimmune diseases.^11–13^ Neutralization of these cytokines is expected to be a potent therapeutic strategy in some autoimmune diseases.^10^ Higher levels of IL-17 and IL-23 expression has been shown in a number of autoimmune disorders.^13^ However, the expression of these cytokines in RHD remains unclear. In this study, we aimed to explore serum levels of IL-17 and IL-23 in RMS patients and compare with those of healthy subjects.

## METHODS

### Study Population

A total of 35 patients admitted to cardiology outpatient clinic between December 2014 and May 2015 who were diagnosed with RMS formed the study group. None of the patients had experienced rheumatic fever (RF) attack within last 12 months. Thirty of 35 RMS patients were on regular benzathine penicilin-G (BP-G) treatment for acute RF prophylaxis. One patient had a penicillin allergy history and 4 were not on prophylaxis regimen because of patient-incompliance. Age- and gender-matched 35 healthy subjects were included as the control group. Patients with heart failure, autoimmune disorders (such as systemic lupus erythematosus, rheumatoid arthritis, inflammatory bowel disease, asthma, or psoriasis), hematologic or rheumatologic disorders, hypertension, diabetes mellitus, acute infection, chronic inflammatory disorders, coronary artery disease, pregnancy, hypersensitivity, pulmonary, renal or hepatic diseases and malignancies were excluded from the study. All patients gave informed written consent before enrollment to the study.

### Serum Collection

Blood samples were collected in citric acid containing tubes and stored at −80 °C after centrifugation. The serum IL-17 and IL-23 levels were measured by using the ELISA kits with respect of the instructions provided by the manufacturer (Sunredbio, Shanghai, PRC).

### Transthoracic Echocardiography

Transthoracic echocardiography was performed in all patients with a commercially available ultrasound system (GE Vivid S5, Vingmed System Five, Horton, Norway) according to the recommendations of the American Society of Echocardiography.^14^

The mitral valve area was measured with both the planimetric and the pressure half time (PHT) methods, and the transmitral gradients were measured with a continuous wave (CW) Doppler in apical 4-chamber view. Following echocardiographic criteria were used to establish the diagnosis of rheumatic mitral stenosis:^15^ mitral valve area ≤ 2 cm^2^, the presence of commissural fusion, leaflet thickening, and alteration of the subvalvular apparatus. Mitral valve anatomy was assessed with the Wilkins score.^16^

### Statistical Analysis

Continuous variables are presented as mean ± standard deviation or median and interquartile ranges, whereas categorical variables are given as number and percentages. The Kolmogorov–Smirnov test was used to verify the normality of the distribution of continuous variables. The independent sample *t* test or the Mann–Whitney *U* test was used for the continuous variables, and the chi-square test was used for categorical variables. Correlation analysis was assessed with the Spearman rank test. Receiver-operating characteristic (ROC) curve analysis was used to determine the optimum cutoff levels of IL-17 and IL-23 that would predict the presence of RMS, and results are shown as odds ratios with 95% confidence intervals (CIs). Statistical analyses were performed using SPSS 18.0 (SPSS Inc, Chicago, IL). *P* value <0.05 was considered as statistically significant.

## RESULTS

There were no differences between groups in terms of age, gender, body mass index (BMI), left ventricular ejection fraction (LVEF), creatinine, hemoglobin, platelet count, and glucose measures (Table 1). The patients with RMS had higher WBC count, high sensitivity C-reactive protein (hsCRP), systolic pulmonary artery pressure (PAPs), left atrial diameter (LAD), IL-17 and IL-23 levels compared to the healthy controls (Table 1).

**TABLE 1:**
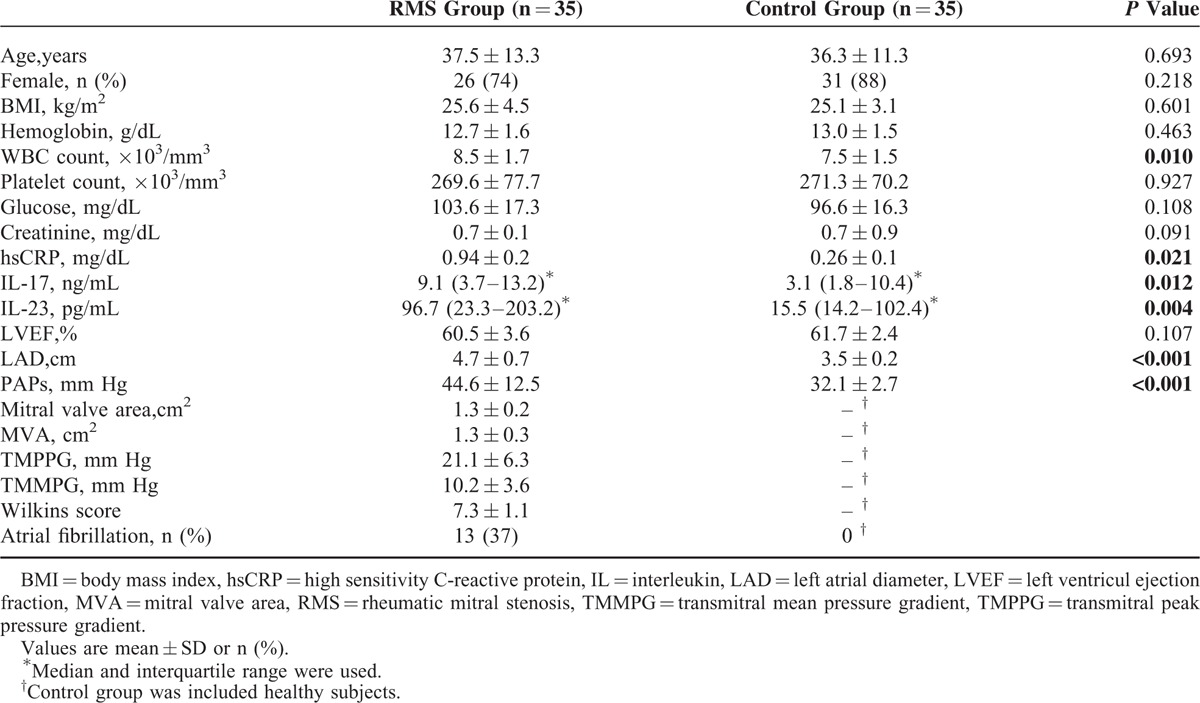
Baseline Demographic, Clinical, and Laboratory Characteristics of the Study Groups

**FIGURE 1:**
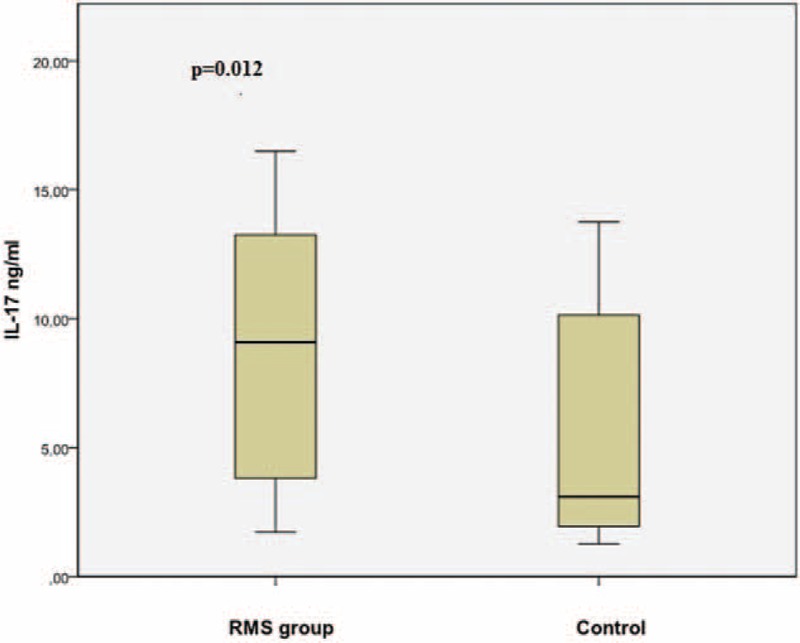
The comparison of serum IL-17 levels between RMS and control group. RMS = rheumatic mitral stenosis.

**FIGURE 2:**
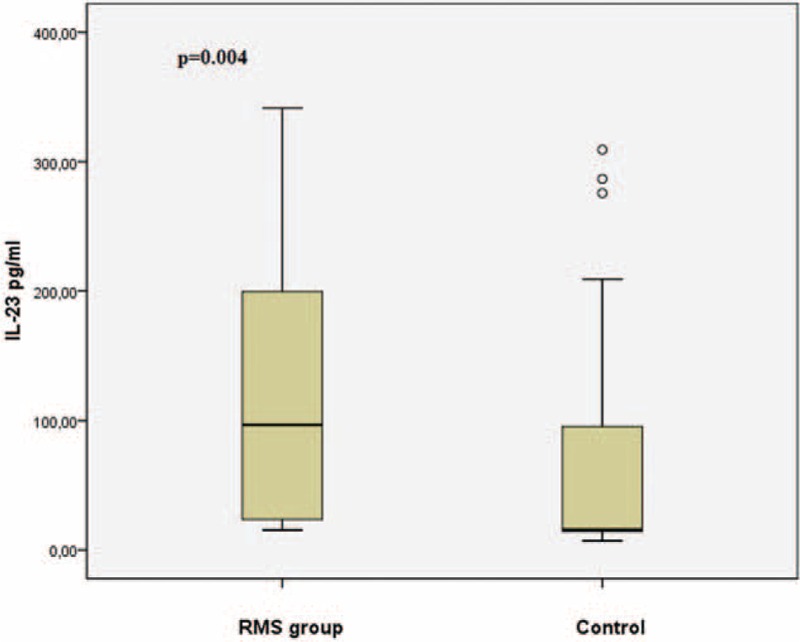
The boxplot graph showing the comparison of serum IL-23 levels between RMS and control group. RMS = rheumatic mitral stenosis.

IL-17 was 9.1 ng/mL (3.7–13.2 ng/mL) in the RMS group and 3.1 ng/mL (1.8–10.4 ng/mL) in the control group (*P* = 0.012) (Figure 1). IL-23 was 96.7 pg/mL (23.3–203.2 pg/mL) in the RMS group and 15.5 pg/mL (14.2–102.4 pg/mL) in the control group (*P* = 0.004) (Figure 2).

Of the 35 patients with RMS, mean MVA was 1.3 ± 0.3 cm^2^, transmitral peak gradient was 21.1 ± 6.3 mm Hg, transmitral mean gradient was 10.24 ± 3.62 mm Hg, left atrial diameter was 4.7 ± 0.7 cm, systolic pulmonary arterial pressure was 44.6 ± 12.5 mm Hg. Mean of the Wilkins score was 7.3 ± 1.1 in the study group. Atrial fibrillation was present in 13 patients.

**FIGURE 3:**
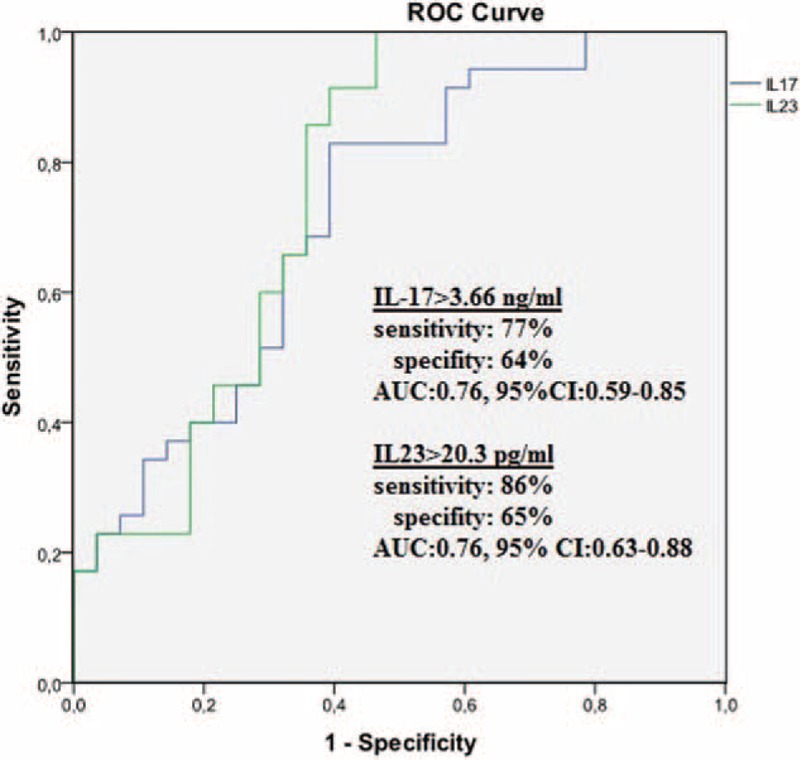
The receiver-operating characteristic (ROC) curve analysis of IL-17 and IL-23 for predicting mitral stenosis. AUC = area under curve, CI = confidence interval.

In the ROC curve analysis, a cut-off value of 3.66 ng/mL for the serum IL-17 level predicted the presence of RMS with a sensitivity of 77% and specificity of 64% (ROC area under curve: 0.723, 95% CI: 0.59–0.85; Figure 3). A cut-off value of 20.3 pg/mL, for serum IL-23 level predicted the presence of RMS with a sensitivity of 86% and specificity of 65% (ROC area under curve: 0.762, 95% CI: 0.63–0.88; Figure 3). Correlation analysis revealed that IL-17 and IL-23 had a significant correlation with hsCRP (*r* = 0.173, *P* = 0.04 and *r* = 0.274, *P* = 0.022, respectively) and LAD (*r* = 0.282, *P* = 0.018 and *r* = 0.428, *P* = 0.01 respectively). Additionally, IL-17 and IL-23 were correlated with each other (*r* = 0.522, *P* < 0.01) (Table 2).

**TABLE 2:**
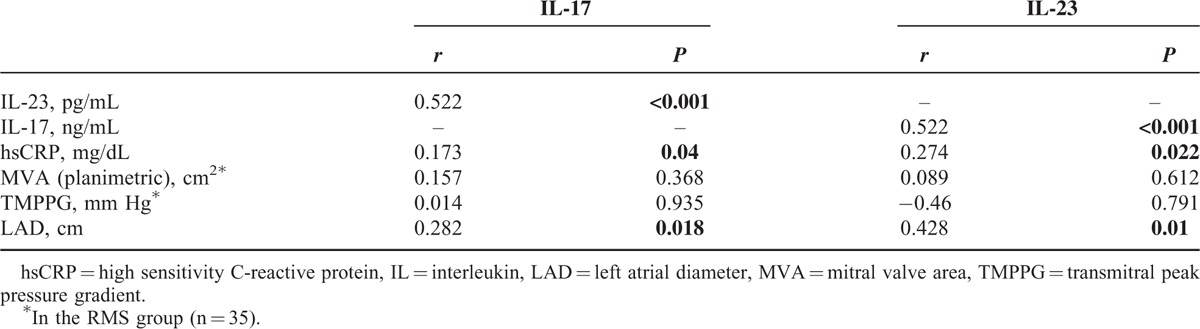
Correlation Analysis for IL-17 and IL-23

## DISCUSSION

In this study, we aimed to compare the serum levels of IL-17 and IL-23 between patients with RMS and healthy subjects. According to our results, serum IL-17 and IL-23 levels were significantly higher in patients with RMS compared to healthy controls. Also, the serum hsCRP level was significantly higher in the patient group. Correlation analysis revealed that IL-17 and IL-23 levels had a significant correlation with hsCRP and LAD. Additionally, IL-17 and IL-23 were significantly correlated with each other.

Rheumatic heart disease is the most serious complication of RF and lead to chronic valvular lesions. Rheumatic mitral valve stenosis is a complication of RHD that leads to significant morbidity and mortality.^2^ RHD still remains an important health burden in many developing countries.^17^

The role of chronic inflammation in RMS has been shown in previous reports. There is a persistent inflammation that causes the damage of the valvular tissue.^8^ There are many studies that investigated the markers of chronic inflammation in RHD patients. Golbasi et al have reported higher serum level of hsCRP in patients with chronic rheumatic valvular disease compared to healthy participants.^18^ In another study that were conducted on patients with RMS, hsCRP levels were significantly higher in patients with RMS, and hsCRP values were correlated with the Wilkins score.^19^

Additionally, in a study by Polat et al reported increased serum levels of hsCRP and Pentraxin-3 as markers of inflammation levels in RMS patients compared to healthy subjects^20^ and Pentraxin-3 was significantly correlated with severity of mitral valve stenosis. Our results were compatible with aforementioned studies as the serum hsCRP levels were significantly higher in our RMS patients compared to healthy subjects. Additionally, we found that IL-17 and IL-23 were significantly correlated with hsCRP.

In another study,^21^ the neutrophyl-to-lymphocyte ratio (NLR) as a recently emerged marker of inflammation was significantly higher in patients with severe RMS when compared to patients with mild to moderate RMS. It was concluded that the NLR may be useful in predicting the presence and severity of RMS.

RMS is a complex disease. Chronic inflammation and the autoimmune reactions constitute the main mechanisms of the pathogenesis. Molecular mimicry, between antigens of the host and GAS, has been thought to be the triggering factor leading to the disease. Both T-cells and crossreactive antibodies have important roles in the cross-recognition between streptococcal antigens and human proteins leading to inflammation and autoimmunity.^22^ In a rat study, Lymbury et al had immunized the rats with pooled synthetic peptides from the conserved C-region of the GAS M5 protein. They demonstrated that there were inflammatory lesions in myocardium and valve tissue by histological examination of cardiac tissue obtained from immunized rats. They suggested that the results were indicated a role for GAS M protein-specific autoreactive T cells in the development of cardiac lesions.^23^

An inflammatory cascade mediates the development of heart lesions with overexpression of a number of inflammatory cytokines including IL-17 and IL-23. Autoreactive CD4 + T cells infiltrate the heart tissue and trigger autoimmune reactions through molecular mimicry.^24^

Although the etiology of RHD is not completely understood, studies have shown that RHD is mediated by humoral and cellular autoimmune responses^4^ and associated with cytokine activities.^8^ T helper 17 (Th17) cells have been recently identified preferential producers of IL-17, IL-17A, IL-17F, IL-21, and IL-22. IL-23 is responsible for the differentiation of Th17 cells from naive CD4 + T cells. IL-17 is a proinflammatory cytokine that is produced by activated T-cells. IL-17 production is increased in response to IL-23 stimulation.^25^ In a study by Bas et al reported that the percentage of peripheral blood Th17 cells and the Th17/regulatuar T cell (Treg) ratio were increased significantly in RHD patients. Th17 and Treg cells play opposite roles in the immune tolerance and autoimmune diseases.^26^ Although Th17 cells and related cytokines have an important effects in defending against various infections, especially extracellular bacterial infections, they have a key role in mediating chronic inflammation and in the development of autoimmune diseases.^11,27^

Previous studies have indicated that Th17 cells and associated cytokines participate in the pathogenesis of various autoimmune disorders such as multiple sclerosis, rheumatoid arthritis, systemic lupus erythematosus, and asthma. The overexpression of Th17-associated cytokines has been shown in some autoimmune diseases.^13^ However, the expression of these cytokines in RMS has not been studied yet. Therefore, in this study, we evaluated serum IL-17 and IL-23 levels in patients with RMS.

The relation between interleukins and chronic inflammation is well known, as well as RHD development. Davutoglu et al have reported that the patients with RMS had increased plasma levels of IL-6, IL-8, IL-2, tumor necrosis factor-alpha (TNFα), and hsCRP as indicators of ongoing inflammation compared with the healthy subjects.^28^

Additionally in a rat study Wen et al have reported that the expression of IL-17, IL-21, IL-6, and IL-23 were significantly increased in mitral valve tissues in rats with RHD compared with normal group and the serum IL-17 and IL-6 concentrations were significantly higher in RHD rats.^13^ They thought that the expression of Th17 cell-associated cytokines is not induced by acute infection of GAS. However, cytokines are overexpressed in the chronic stable stage of RHD, so the autoimmune injury induced by Th17 cells was thought to be a key factor.

The actions of IL-17 and IL-23 in autoimmune diseases make them important therapeutic targets in autoimmune disorders.^10^ Previous studies have shown that blocking TNFα, IL-6, IL-23, IL-17, or their corresponding receptors by using the neutralizing antibodies is highly effective in the treatment of some autoimmune diseases such as psoriasis, rheumatoid arthritis, and inflammatory bowel disease.^9,27^ Additionally, new antibody drugs targeting IL-17A, IL-17RA, IL-17F, IL-17A/TNF, IL-23 are being tested in psoriasis, psoriatic arthritis, ankylosing spondylitis, rheumatoid arthritis, autoimmune uveitis, asthma, and multiple sclerosis. The antibody drugs that neutralize IL-23 or IL-17A have shown a significant efficacy in the treatment of psoriasis. These agents also show hopeful results in ankylosing spondylitis and multiple sclerosis.^27^

There is yet no specific treatment to prevent the progression of RHD. Primary prevention of acute RF consists of early diagnosis and treatment of GAS tonsillopharyngitis with penicillin.^29^ In patients with a prior episode of RF, secondary prevention is critically important. Recurrent pharyngeal GAS infection can trigger a severely exaggerated immune response in these patients and the recurrent RF is associated with a higher incidence of carditis.^30^ Secondary prevention with intramuscular injection of BP-G every 3 to 4 weeks is stil recommended,^31^ but the efficacy of secondary prevention is limited in prevention of RHD progression; however, the compliance of patients with BP-G treatment is relatively low.^32^ For this reason, new strategies and therapies are needed to prevent the relapse of acute RF and the progression of RHD.

Neutralizing inflammatory cytokines or antagonizing their receptor function has been considered as a useful therapeutic strategy to treat autoimmune diseases. In this respect, new therapies targeting IL-17 and IL-23 and their reseptors as studied in some autoimmune diseases may promise a new approach for patients with RHD.

To our best knowledge, this is the first study to evaluate serum levels of IL-17 and IL-23 in patients with RMS.

### Study Limitations

Relatively small sample size was the main limitation of our study. Another limitation was the absence of other associated cytokines. The findings cannot be generalized to overall population. These results need to be confirmed by multicentre studies with larger sample sizes.

## CONCLUSION

We demonstrated that serum levels of IL-17 and IL-23 are significantly higher in patients with RMS compared to those of healthy subjects. IL-17 and IL-23 expression may have a possible role in inflammatory processes that result in RMS development. Further large-scale studies are required to confirm our results.
